# Lesion size and cystic morphology are key determinants of parathyroid hormone washout in primary hyperparathyroidism

**DOI:** 10.3389/fendo.2025.1654110

**Published:** 2025-09-16

**Authors:** İlker Çordan, Oguzhan Aksu

**Affiliations:** Division of Endocrinology and Metabolism, Department of Internal Medicine, Konya City Hospital, Hamidiye School of Medicine, University of Health Sciences, Konya, Türkiye

**Keywords:** primary hyperparathyroidism, parathyroid hormone washout, ultrasonography, biopsy, fine-needle, parathyroid adenoma, ectopic tissue

## Abstract

**Purpose:**

Accurate preoperative localization of parathyroid adenomas is of great importance to ensure targeted and effective surgical treatment of primary hyperparathyroidism (PHPT). In this context, parathyroid fine-needle aspiration biopsy (FNAB) performed under ultrasound guidance plays a crucial role. This study aimed to evaluate the relationship between ultrasonographic features of parathyroid adenomas and parathyroid hormone washout (PTH-WO) values.

**Methods:**

Between January 2022 and August 2024, data from 128 parathyroid adenomas in 122 patients who were prepared for surgery due to PHPT and underwent ultrasound-guided FNAB along with PTH-WO testing were retrospectively evaluated. The obtained PTH-WO results were compared with B-mode and Doppler ultrasound findings.

**Results:**

In adenomas with a long axis greater than 10 mm and those containing cystic components, PTH-WO levels were found to be significantly higher (p = 0.005 and p = 0.02, respectively). Additionally, a positive correlation was identified between PTH-WO levels and the short and long axis dimensions of the adenomas. In contrast, no significant relationship was observed between PTH-WO levels and B-mode (grayscale) or Doppler (vascularity) ultrasound features.

**Conclusion:**

PTH-WO values vary according to the ultrasonographic dimensions of parathyroid adenomas and are measured at higher levels in cystic adenomas. Other ultrasonographic features do not significantly affect PTH-WO levels. Therefore, the size of the lesion should be taken into account when interpreting PTH-WO results.

## Introduction

Primary hyperparathyroidism (PHPT) is a common endocrine disorder characterized by hypercalcemia resulting from autonomous parathyroid hormone (PTH) secretion. The most frequent cause is parathyroid adenomas, and the standard treatment is parathyroidectomy (1). A fundamental prerequisite for surgical success is the accurate preoperative localization of the hyperfunctioning adenoma (2).

Neck ultrasonography and Tc-99m sestamibi scintigraphy are typically the first-line imaging modalities; however, both have limitations. Coexisting conditions, e.g., thyroid nodules, lymph nodes, and chronic lymphocytic thyroiditis, may lead to false-positive results (3, 4). In such cases, minimally invasive techniques such as parathyroid fine-needle aspiration biopsy (FNAB) and parathyroid hormone washout (PTH-WO) can support accurate localization. PTH-WO is based on the measurement of PTH levels in the washout fluid obtained from suspected lesions under ultrasound guidance. This method has high specificity and provides a complementary diagnostic contribution to surgical planning (5). However, there is no established diagnostic threshold for this method, and morphological factors such as adenoma size, location, and vascularity that may affect its accuracy have not yet been clearly defined (6).

This study evaluated the relationships between PTH-WO levels and the size, location, morphological characteristics, B-mode and Doppler ultrasonographic findings, and demographic data of parathyroid adenomas in patients diagnosed with PHPT. The aim was to identify the clinical and morphological factors affecting PTH-WO levels, thereby improving the preoperative diagnostic accuracy of the method and contributing to clinical decision-making processes.

## Methods

This retrospective cross-sectional study included data from patients diagnosed with PHPT between January 2022 and August 2024 in a tertiary care center. The FNAB and PTH-WO results of 128 parathyroid adenomas from 122 patients evaluated for preoperative localization were retrospectively analyzed. Demographic, clinical, laboratory, and imaging data were obtained from the hospital’s medical record system.

### Patient selection

**Figure 1:**
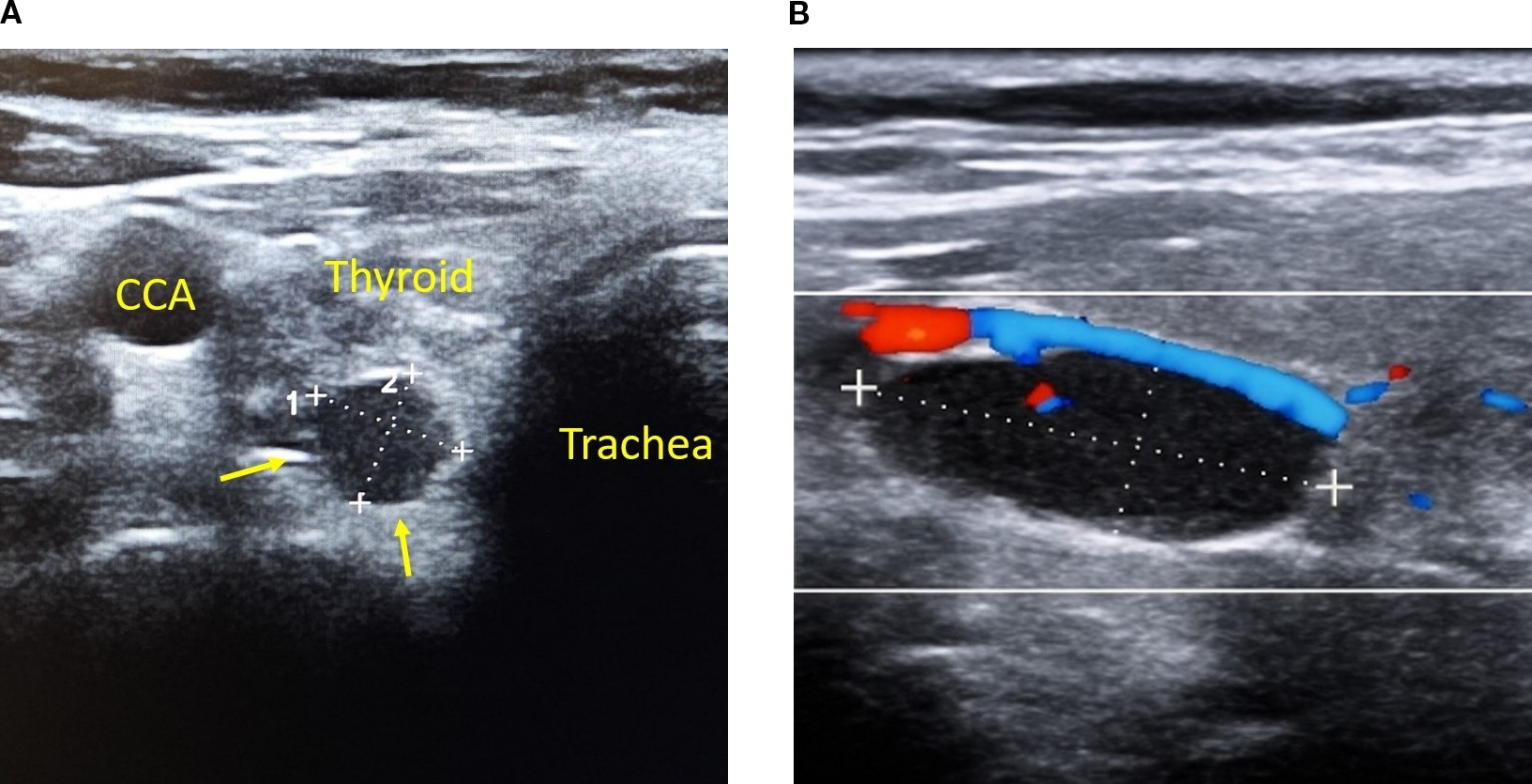
Typical ultrasonographic appearance of a parathyroid adenoma. **(A)** A hypoechoic, well-circumscribed lesion (yellow arrows) adjacent to the posterior aspect of the thyroid gland represents a right inferior parathyroid adenoma. Note the proximity to the common carotid artery (CCA) and trachea, labeled for anatomical reference. The lesion is located in a typical orthotopic position and demonstrates characteristic features of parathyroid adenomas on B-mode ultrasonography, including an oval shape and well-defined margins. **(B)** A well-defined, hypoechoic lesion representing a parathyroid adenoma is shown on color Doppler imaging. Doppler evaluation reveals a characteristic polar vascularization pattern, with feeding vessels entering from the superior pole and forming a peripheral vascular arc around the lesion. This vascular pattern is commonly observed in parathyroid adenomas and helps differentiate them from adjacent thyroid nodules and cervical lymph nodes, which typically exhibit different vascular profiles.

**Figure 2:**
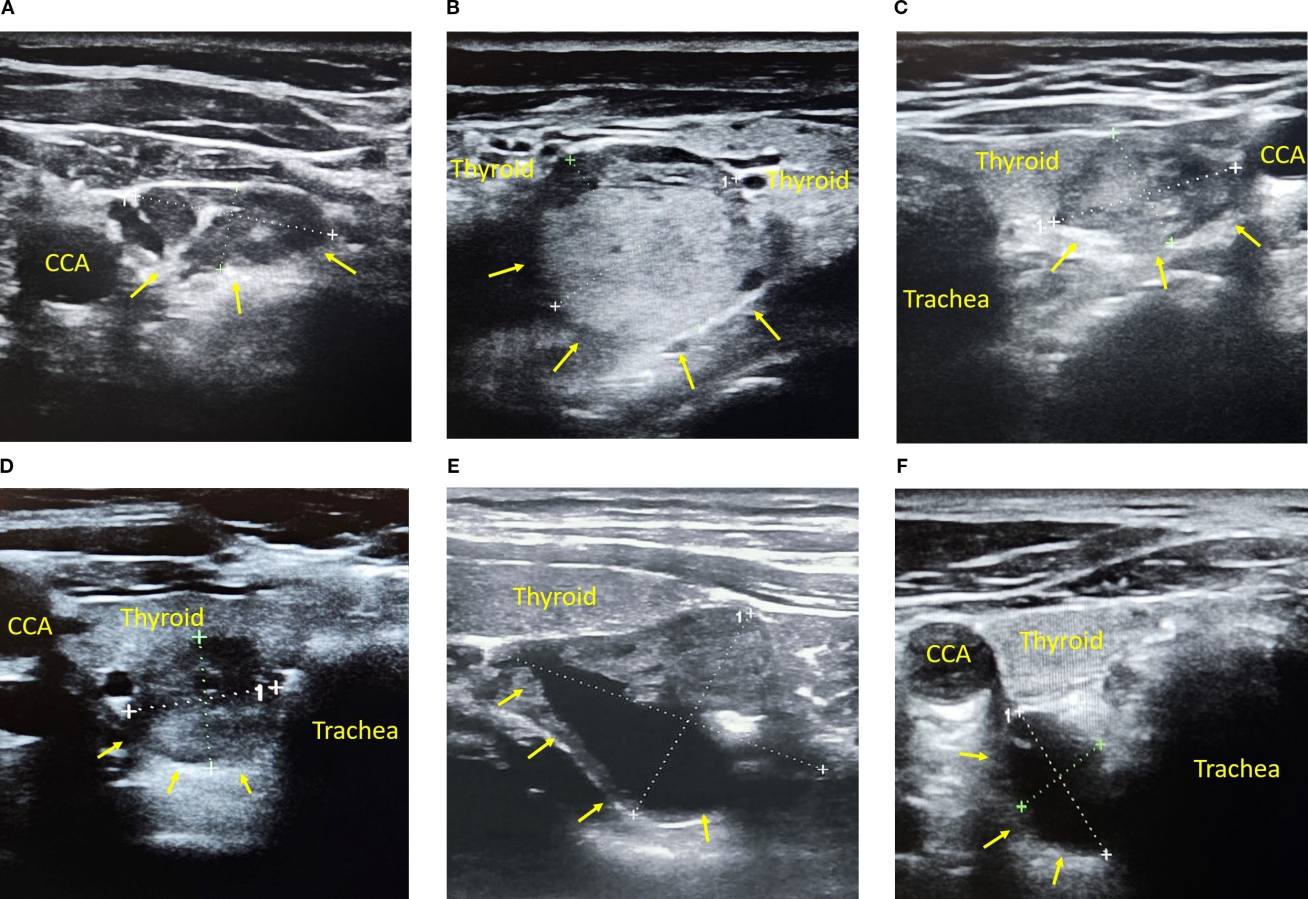
Atypical parathyroid adenomas diagnosed via PTH-WO **(A–F)**. **(A)** Right inferior parathyroid adenoma (18×7 mm) adjacent to the thyroid, featuring a hyperechoic hilum-like area, mimicking a lymph node. **(B)** Large parathyroid adenoma (20×20 mm) with hyperechoic echotexture, resembling a thyroid nodule in Hashimoto’s thyroiditis. **(C)** Right inferior intrathyroidal parathyroid adenoma (17×13 mm), fully embedded within the thyroid parenchyma and challenging to distinguish from a thyroid nodule. **(D)** Left inferior intrathyroidal parathyroid adenoma (14×12 mm) with indistinct margins, mimicking an atypical thyroid nodule. **(E)** Predominantly cystic parathyroid adenoma (32×22 mm) lacking vascularity and negative on scintigraphy; diagnosed via PTH-WO. **(F)** Purely cystic parathyroid adenoma (15×9 mm) between the trachea and CCA; anechoic, avascular, and scintigraphy-negative; confirmed by PTH-WO. *Note: Yellow arrows indicate the adenomas. CCA, common carotid artery; PTH-WO, parathormone washout*.

The study included patients over the age of 18 years who were diagnosed with PHPT according to current guidelines (1), had surgical indications, and underwent PTH-WO due to inconclusive or non-diagnostic findings on preoperative ultrasonography and/or sestamibi SPECT and whose diagnoses were confirmed surgically. Ultrasound images of ambiguous adenoma variants and classic parathyroid adenomas are presented in **Figures 1** and **2**.

The exclusion criteria were diagnoses of secondary or tertiary hyperparathyroidism, suspected parathyroid hyperplasia due to familial syndromes, end-stage renal disease, pregnancy, and incomplete or insufficient clinical data. Patients meeting these criteria were excluded from the study.

### Imaging methods and PTH-WO analysis

For preoperative localization, all patients underwent B-mode (grayscale) and Doppler ultrasonography using a 12-MHz linear array probe on a Mindray DC-60 Exp HD (Shenzhen, China) ultrasound device. Prior to the PTH-WO procedure, the ultrasonographic features of the adenomas were recorded in the electronic patient registry system.

FNAB was performed under real-time ultrasound guidance using a free-hand technique with a 23-G needle attached to a 10 mL syringe and negative pressure. The aspirate obtained was diluted by washing at least three times in a plastic tube containing 1 mL of saline and delivered to the laboratory within 10 minutes. Due to the low sensitivity of cytological evaluation, all analyses were performed on the aspirate washout fluid (7).

PTH levels in plasma and aspirate washout fluid were measured using Cobas e411 and Immulite XPi immunoassay analyzers with a measurement range of 3–5,000 pg/mL. In our laboratory, the reference range for plasma PTH was set at 15–65 pg/mL. Only patients whose diagnoses were confirmed through biochemical remission following surgery were included in the study. The minimum value accepted for PTH-WO was 33 pg/mL, based on the lowest positive PTH-WO level confirmed surgically in our series.

### Statistical analysis

For numerical variables, means and standard deviations or medians (Q1–Q3) were reported, while for categorical variables, frequencies and percentages were presented. The chi-square or Fisher’s exact test was used for the analysis of categorical variables, and the t-test or analysis of variance was used for numerical variables. Relationships between numerical variables were evaluated using Spearman correlation coefficients. Boxplot graphs were presented for PTH-WO values, and linear regression analysis was performed to examine the relationships. All analyses were conducted using R version 4.4.2 (R Core Team, 2024), and a p-value of <0.05 was considered statistically significant.

## Results

The study included a total of 122 patients, of whom 90 were female (73.8%) and 32 were male (26.2%), resulting in a female-to-male ratio of 2.8:1. The mean age was calculated to be 53.7 years (range: 18–84 years).

A single adenoma was detected in 95.1% (n = 116) of the patients, while double adenomas were present in 4.9% (n = 6), resulting in a total of 128 parathyroid adenomas analyzed. The mean plasma PTH level was 145.43 ± 102.59 pg/mL, and the median PTH-WO value was 1,698.50 pg/mL (512.25–5,000.0). The demographic, clinical, and laboratory characteristics of the patients are presented in **Table 1**.

**Table 1:** Demographic, clinical, and laboratory characteristics of patients with primary hyperparathyroidism (n = 122).

Variable	Mean ± SD
Age (years)	53.37 ± 12.28
Sex^1^	
Male	32 (26.23%)
Female	90 (73.77%)
Height (cm)	159.25 ± 8.70
Weight (kg)	78.84 ± 13.86
BMI (kg/m²)	31.16 ± 5.32
Calcium (mg/dL)	11.14 ± 0.64
Parathyroid hormone (pg/mL)	145.43 ± 102.59
Phosphorus (mg/dL)	2.71 ± 0.59
Vitamin D (ng/mL)	17.19 ± 9.44
Creatinine (mg/dL)	0.73 ± 0.19
Albumin (g/dL)	45.54 ± 3.21
Alkaline phosphatase (U/L)	105.08 ± 40.44
24-hour urinary calcium (mg)	377.25 ± 166.65
PTH-WO (pg/mL) (128 adenomas)2	1,698.50 (512.25-5,000.0)

^1^Presented as number (percentage). ^2^Presented as median (25^th^–75^th^ percentile).

SD, standard deviation; BMI, body mass index; PTH-WO, parathyroid hormone washout.

The mean long axis of the adenomas was measured as 15.51 ± 6.85 mm (range: 6–36 mm), and it was ≤10 mm in 27.34% (n = 35) of the patients, 11–15 mm in 32.81% (n = 42), and >15 mm in 39.84% (n = 51). Regarding localization, 84.4% (n = 108) of the patients had orthotopic and 15.6% (n = 20) had ectopic adenomas. Among orthotopic adenomas, the most frequent location was the right inferior, while the right superior was the least common. In the distribution of ectopic adenomas by sex, ectopic parathyroid adenomas were detected in 45% (n = 9) of the males and 12.22% (n = 11) of the females, with a significantly higher prevalence in the former (p = 0.032).

In ultrasonographic examination, B-mode imaging revealed that most adenomas were hypoechoic, well-defined, and solid lesions. In Doppler imaging, polar vascularity was the most common finding, whereas diffuse vascularity was the rarest. Data on the size, localization, and ultrasonographic features of the parathyroid adenomas are presented in **Table 2**.

**Table 2:** B-Mode and doppler ultrasonography features of parathyroid adenomas (n = 128).

Variable	n (%)
Localization	
Orthotopic adenomas	
Left inferior	29 (22.66%)
Left superior	23 (17.97%)
Right inferior	34 (26.56%)
Right superior	22 (17.19%)
Ectopic adenomas	20 (15.63%)
Adenoma dimensions^1^	
Long diameter (mm)	15.51 ± 6.85
Short diameter (mm)	7.55 ± 2.99
Short-to-long axis ratio (shape index)	0.52 ± 0.16
B-Mode Features	
Hypoechoic	77 (60.2)
Mixed echogenicity	51 (39.8)
Irregular border	46 (36)
Bilobed shape	19 (14.8)
Cystic component	30 (23.4)
Doppler Features	
Polar vascularity	100 (78.13)
Vascular arc	51 (39.84)
Diffuse vascularity	11 (8.6)

^1^Presented as mean ± standard deviation.

Compared to ultrasonographically measured dimensions, PTH-WO values were significantly higher in adenomas with a long axis of >10 mm and a short axis of >5 mm (p = 0.005 and p = 0.008, respectively).

The Spearman correlation analysis revealed that PTH-WO values increased with adenoma size. A positive and significant correlation was observed between the long axis and PTH-WO (r = 0.29, p < 0.001). Similarly, PTH-WO had a positive correlation with the short axis (r = 0.28, p < 0.01). However, the relationship with the long axis was more significant.

The short-to-long axis ratio (shape index) was calculated as a mean of 0.52 ± 0.16. The median PTH-WO value was 1,746.0 pg/mL (623.0–4,503.0) in the group with an aspect ratio of ≤0.5 and 1,428.0 pg/mL (487.5–5000.0) in the group with a ratio of >0.5, indicating no statistically significant difference (p = 0.72). Moreover, analyses using different cut-off values for the shape index (0.4, 0.6, and 0.7) yielded no significant differences (p = 0.51, p = 0.99, and p = 0.65, respectively). Lastly, no significant correlation was found between the shape index and PTH-WO (r = -0.04, p > 0.05).

**Figure 3:**
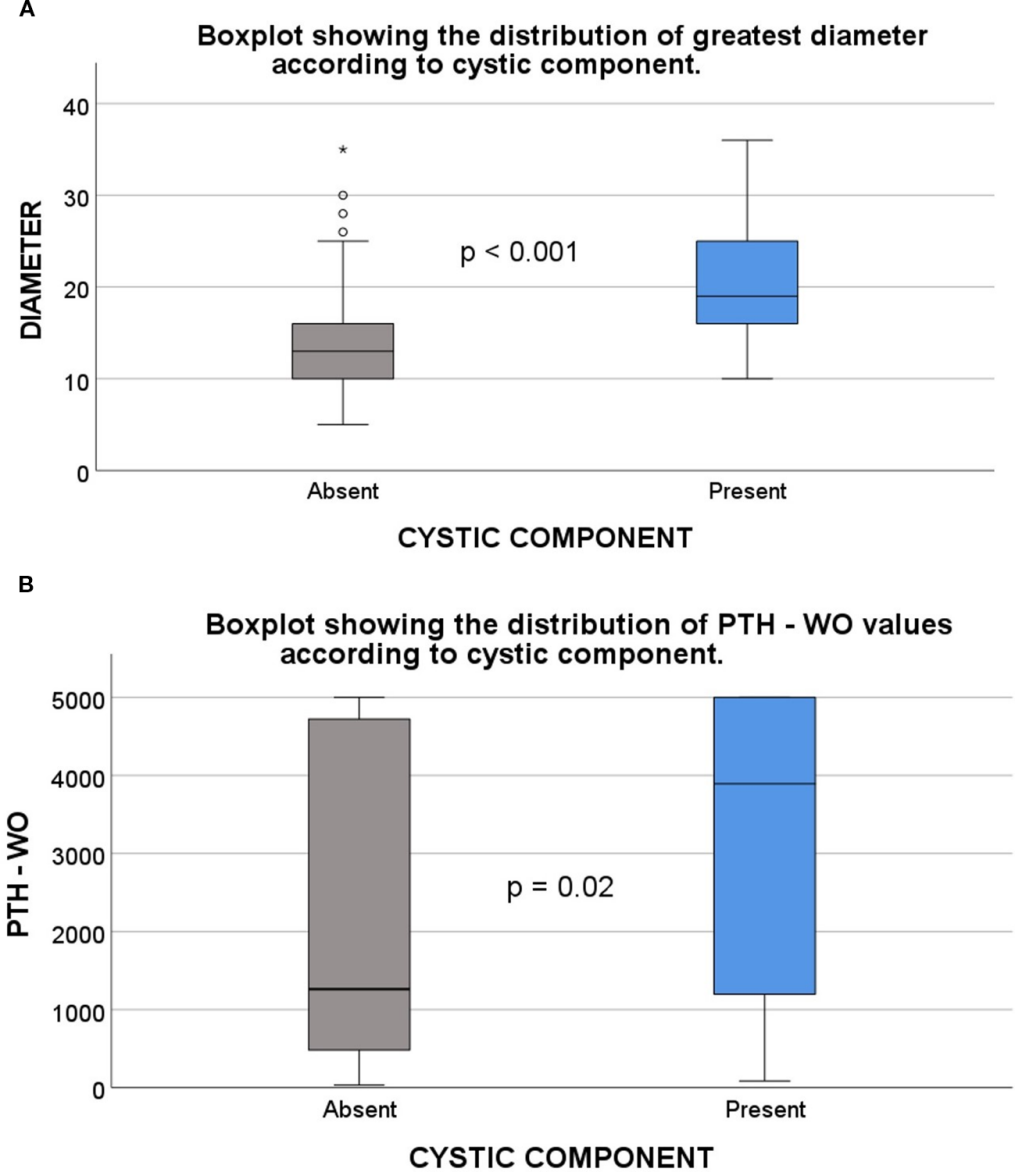
Comparison of cystic and solid parathyroid adenomas based on lesion size and PTH-WO levels. **(A)** Box plot showing that parathyroid adenomas with cystic components have significantly larger diameters than solid adenomas (p < 0.001). Lesion diameter was measured as the greatest axis on ultrasonography. **(B)** Box plot comparing PTH-WO values between cystic and solid adenomas. Cystic adenomas demonstrated significantly higher PTH-WO levels (p = 0.02). *Note: “Present” refers to adenomas with a cystic component, while “Absent” refers to solid adenomas. PTH-WO, Parathormone washout*.

In the analysis based on the presence of cystic components, cystic adenomas (24.57 ± 7.38 mm) were found to be significantly larger than solid adenomas (13.74 ± 5.75 mm) (p < 0.001). In addition, PTH-WO values in cystic adenomas were significantly higher than those in solid adenomas (p = 0.02) (**Supplementary Table S1**). The distribution of cystic and solid parathyroid adenomas according to size and PTH-WO values is presented in a box plot in **Figure 3**.

After excluding cystic adenomas, the significant relationship between the PTH-WO value and adenoma size persisted in solid parathyroid adenomas (n = 98). Among solid adenomas with a long axis of ≤10 mm, the median PTH-WO value was 774 pg/mL (326.50–1,686.25), while it was 2,520 pg/mL (522.75–5,000) for those with a long axis of >10 mm (p = 0.037), and the difference was statistically significant. For the short axis, PTH-WO values were 815 pg/mL (339–1,617) in adenomas ≤ 5 mm and 2,448 pg/mL (493–5,000) in those > 5 mm, again showing a significant difference (p = 0.048).

Although PTH-WO values were higher in males and in adenomas located ectopically, these differences were borderline significant (p = 0.07 and p = 0.077, respectively). No significant relationship was found between PTH-WO values and age, body mass index (BMI), echogenicity, margin regularity, number of lobes, or vascular findings in Doppler ultrasonography (p > 0.05). A detailed comparison of PTH-WO values by clinical and ultrasonographic parameters is provided in **Supplementary Table S1**.

**Figure 4:**
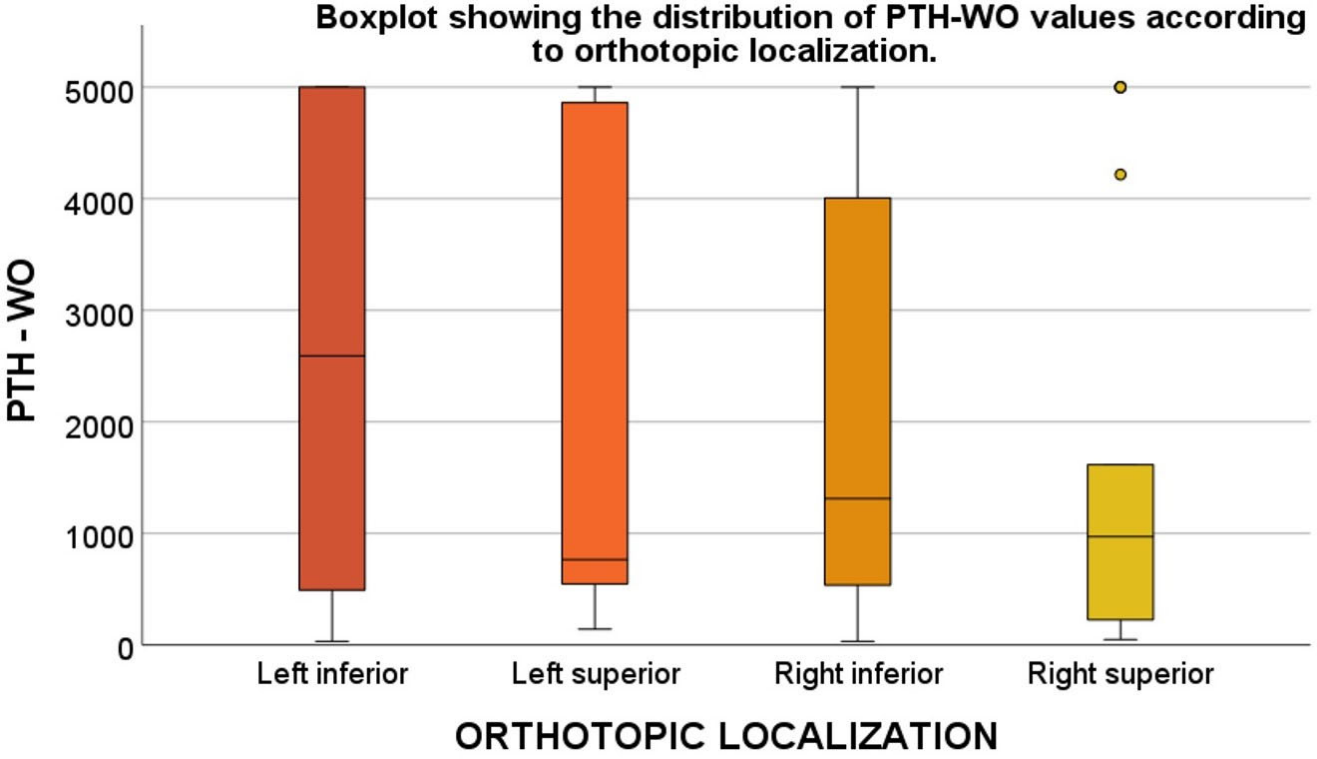
Distribution of PTH-WO values by orthotopic parathyroid adenoma location. Box plot comparing PTH-WO values across orthotopic adenoma locations: left inferior, left superior, right inferior, and right superior. Left inferior adenomas exhibited the highest PTH-WO levels, while right superior adenomas had the lowest median values. *Note: “Orthotopic localization” refers to adenomas in typical anatomical positions relative to the thyroid* gland. *PTH-WO: Parathormone washout*.

In orthotopic parathyroid adenomas, PTH-WO values were 2592.0 pg/mL (493.0–5,000.0) in the left inferior region, 767.0 pg/mL (549.0–4,861.5) in the left superior, 1312.5 pg/mL (558.5–3,849.75) in the right inferior, and 971.0 pg/mL (250.75–1,563.25) in the right superior. Although values in the left inferior region were higher, no statistically significant difference was observed among the four locations (p = 0.24). The distribution of PTH-WO values by localization is shown in a box plot in **Figure 4**.

When parathyroid adenoma size was compared according to plasma PTH levels and clinical and ultrasonographic variables, significant relationships were observed between adenoma size and mixed echogenicity (p < 0.001), irregular borders (p = 0.005), cystic components (p < 0.001), and presence of vascular arcs (p < 0.001). In terms of plasma PTH levels, significant differences were observed only with male sex (p = 0.048) and presence of cystic components (p = 0.002), with no significant associations found for other variables (p > 0.05).

A moderate positive correlation was identified between adenoma size and plasma PTH (ρ = 0.36, p < 0.001). The correlation between plasma PTH and PTH-WO levels was weak but significant (ρ = 0.19, p = 0.035).

In linear regression analysis for variables associated with PTH-WO, the long axis, short axis, BMI, and presence of cystic components were found to have significant associations (**Table 3**). According to multivariate linear regression analysis evaluating variables potentially affecting PTH-WO values, only the long axis of the adenoma emerged as a statistically significant predictor (β = 55; 95% confidence interval [CI]: 0.04–111; p = 0.05). Male sex (β = 602; 95% CI: -194–1398; p = 0.13), BMI (β = -35; 95% CI: -101–31; p = 0.30), and presence of cystic components (β = 524; 95% CI: -367–1415; p = 0.24) did not have independent and significant effects on PTH-WO levels. The explanatory power of the model was low, with a coefficient of determination R² of 0.117.

**Table 3:** Univariable linear regression analysis of parathyroid hormone washout.

Variable	Beta (95% CI)	p-value
Age	13 (-16, 41)	0.39
Male	780 (-5.4, 1,565)	0.052
Long axis diameter	78 (30, 126)	**0.002**
Short axis diameter	148 (45, 251)	**0.005**
Body mass index	-67 (-130, -2.9)	**0.041**
Ectopic	903 (-44, 1,851)	0.062
Mixed echogenicity	2 (-710, 715)	0.99
Irregular borders	442 (-281, 1164)	0.23
Cystic component	992 (188, 1797)	**0.016**
Polar vascularity	-536 (-1,375, 302)	0.21
Vascular arc	439 (-270, 1147)	0.22

CI, confidence interval. Bold values denote statistically significant results (p < 0.05).

## Discussion

Preoperative localization of parathyroid adenomas is a critical factor that directly affects the success of PHPT surgery. Parathyroid FNAB and PTH-WO are effective and complementary diagnostic tools that support preoperative localization, especially in cases where imaging methods prove insufficient. In our study, the relationship between the ultrasonographic features of 128 parathyroid adenomas and PTH-WO levels was evaluated, and the factors affecting the diagnostic accuracy of this method were investigated. To our knowledge, this study is among the first to systematically assess the ultrasonographic factors affecting PTH-WO levels.

Our study demonstrated that the ultrasonographic dimensions of parathyroid adenomas were consistent with PTH-WO levels. In particular, larger and cystic adenomas were associated with higher PTH-WO values. Although ectopic adenomas exhibited higher PTH-WO levels compared to juxtathyroidal adenomas, this difference was on the borderline of statistical significance. No significant correlation was found between PTH-WO levels and B-mode (margin regularity, echogenicity, and lobulation) or Doppler ultrasonographic findings. In regression analyses, short axis, long axis, and cystic component were found to be influential; however, in multivariate analysis, only the long axis remained an independent determinant. The findings of our study indicate that PTH-WO levels are primarily associated with adenoma size and that cystic parathyroid adenomas, compared to solid adenomas, have significantly larger dimensions and higher PTH-WO levels. Other ultrasonographic parameters appear to have limited effect on PTH-WO levels.

With a sensitivity of 70–100% and specificity of 75–100%, PTH-WO stands out as a more reliable localization method compared to scintigraphy and ultrasonography due to its ability to provide biochemical confirmation (5). In our study, the median PTH-WO level was 1,698.50 pg/mL, which is consistent with values reported by Gökçay Canpolat et al. (1,824 pg/mL) and Güneş et al. (1,600 ng/L) (5, 8). PTH-WO values exceeding plasma PTH levels contribute significantly to diagnosis; however, this elevation lacks additional clinical significance beyond diagnostic confirmation. Establishing a reliable lower threshold value is important to avoid false negatives, but high plasma PTH levels in PHPT and the risk of contamination complicate this determination. In the literature, proposed PTH-WO thresholds range from 20 to 1,000 ng/L (6). While Kwak et al. and Kuzu et al. considered PTH-WO levels higher than serum PTH sufficient for positivity (9, 10), other researchers regarded the PTH-WO/plasma PTH ratio as more significant (11). In our own series, although plasma PTH levels were higher in male patients, only a borderline significance was found between sexes in terms of PTH-WO values. Additionally, a weak correlation was observed between PTH-WO and plasma PTH levels, and even in cases with double adenomas having identical plasma PTH levels, significant differences in PTH-WO values were noted. These findings suggest that relying solely on plasma PTH levels may be inadequate and unreliable when interpreting PTH-WO results.

Sacks et al. (12) suggested that, apart from blood contamination, no other tissues contain PTH and recommended accepting PTH-WO levels above 20 pg/mL as positive. Güneş et al. (8) demonstrated minimal systemic influence by detecting low PTH-WO levels (14.0 ng/L) in samples obtained from thyroid nodules in patients with PHPT. Similarly, another study observed no false positives for PTH-WO levels up to 20% of plasma PTH (13). In the current study, 6.25% of cases had PTH-WO levels lower than plasma PTH, and the lowest positive value was 33 pg/mL. This suggests that the test can still provide diagnostic value even at low PTH-WO levels. For all these reasons, we consider it necessary to establish an absolute PTH-WO threshold value that is independent of plasma PTH levels in order to avoid false negatives and ensure diagnostic reliability. The clonal heterogeneity of parathyroid adenomas reflects the complex etiopathogenesis and clinical variability of PHPT (14). In addition to these biological differences, variations in adenoma size and location may also cause significant variability in PTH-WO results. Therefore, to maximize the diagnostic accuracy of the PTH-WO test, we believe that it is essential to perform a holistic evaluation of morphological and biological characteristics, such as the embryological origin, vascularity, and anatomical relationship of the adenoma to surrounding tissues.

PHPT is a disease that varies with age and sex and is particularly more common in older individuals and postmenopausal women (15). The data obtained from our patient series also support this observation. The literature on the effects of age and sex on PTH-WO is limited. This may be related to the lower prevalence of PHPT in men and the typically small sample sizes in PTH-WO-related studies. Current data suggest that sex does not have a significant impact on PTH-WO levels (16). On the other hand, it is known that plasma PTH levels are higher in men compared to women (17), a finding also confirmed by our study. Although adenoma sizes were similar, plasma PTH levels were higher in men; however, the increase in PTH-WO levels showed only borderline statistical significance. Nonetheless, the more frequent occurrence of ectopic adenomas in male patients suggests that biological or anatomical sex-based differences may affect PTH-WO levels.

The positive relationship between adenoma size and plasma PTH levels in PHPT cases is well known (18). However, the relationship between the PTH-WO level and adenoma size remains controversial in the literature. In this study, adenomas with long axis > 10 mm and short axis > 5 mm had higher PTH-WO levels and showed positive correlation with both dimensions. This finding is consistent with the results of Popowicz et al., who reported a correlation between lesion volume and PTH levels when small and deeply located lesions were excluded (13). On the other hand, some studies have failed to confirm this relationship and have even reported higher PTH-WO levels in smaller adenomas (11, 19, 20). These contradictory findings may be attributed to the technical limitations of the PTH-WO test. A narrow short axis, deep localization, and the presence of multinodular goiter can reduce aspiration efficiency (11, 13). While the PTH-WO test is generally considered safe (21) and no major complications were observed in our study the procedure may be repeated in cases of inconclusive results; however, long-term risks such as fibrosis and parathyromatosis in the surgical field must be considered (22, 23). In this context, standardized protocols that define reliable lower threshold values and take into account technical challenges and adenoma size should be developed.

One of the major diagnostic challenges in PHPT is the accurate differentiation of cystic parathyroid adenomas. These lesions may resemble lymph nodes or thyroid nodules on imaging, which may lead to diagnostic confusion. In this variant, the accuracy of sestamibi SPECT alone for preoperative localization is limited (29%), but this rate can increase to 79% when combined with cervical ultrasonography (24). Therefore, the PTH-WO test, performed under ultrasound guidance, offers additional diagnostic value through biochemical confirmation. In our study, cystic adenomas were found to be significantly larger and to exhibit higher PTH-WO levels compared to solid adenomas. This may be related to increased lesion activity, accumulation of PTH in cystic fluid, or embryological differences (25). However, in multivariate analyses, only lesion size remained an independent determinant of PTH-WO levels. Even after excluding these lesions, the significant relationship between lesion size and PTH-WO levels persisted in solid adenomas, which further supports the determining role of size. In contrast, the limited explanatory power of the model (R² = 0.117) suggests that other biological or technical factors may also influence PTH-WO levels.

While imaging of parathyroid adenomas is not mandatory for diagnosing PHPT, it important for surgical planning to evaluate the orthotopic or ectopic position of the adenoma and its anatomical relationship with the thyroid. Orthotopic adenomas typically arise from one of the four parathyroid glands located posterior to the thyroid, although gland number can vary among individuals (26). In our study, among orthotopic adenomas, the most common location was the right inferior, and the least common was the right superior, consistent with the literature (27) and reflecting the effect of embryological development on anatomical localization. Superior glands originate from the fourth pharyngeal pouch, while inferior glands arise from the third and migrate a longer and more variable course with the thymus (28, 29). However, these embryological differences did not appear to have a clinically significant effect on PTH-WO levels. Although PTH-WO levels were higher in left inferior adenomas, this difference was not statistically significant. Therefore, we conclude that in orthotopic parathyroid adenomas, PTH-WO levels are independent of anatomical location and embryological origin.

Ectopic parathyroid glands may be located anywhere from the base of the tongue to the mediastinum, and their atypical positions pose challenges in diagnosis and treatment (29, 30). The PTH-WO test contributes diagnostically by providing biochemical confirmation in distinguishing ectopic adenomas from similar-appearing cervical lesions. In our study, the frequency of ectopic adenomas was 15.6%, similar to the literature. However, since only regions accessible to ultrasonography were evaluated, this rate may appear elevated compared to the general PHPT population (31). This finding is consistent with the fact that our study population consisted of cases with difficult localization on imaging. The more frequent occurrence of ectopic adenomas in male patients was notable, suggesting that this possibility should be considered in the differential diagnosis, especially in male patients with inconclusive imaging findings. Although ectopic adenomas were similar in size to orthotopic adenomas, PTH-WO levels were found to be borderline significantly higher. This increase may be due to technical reasons such as more effective aspiration due to proximity to the skin, biological factors such as embryological origin, anatomical differences, or sex-specific physiological effects. Whether these differences are due to technical or biological/anatomical factors should be clarified through more comprehensive studies.

Despite its limitations, ultrasonography remains a primary tool for the evaluation of parathyroid lesions. Typically, an enlarged parathyroid gland appears as a well-defined, oval-shaped, homogeneously hypoechoic structure on B-mode imaging. However, some adenomas may present as isoechoic, heterogeneous, or cystic (32). Age-related fat accumulation and degenerative changes can increase echogenicity and complicate diagnosis. Therefore, the proportion of non-hypoechoic adenomas can reach up to 27% (33, 34). In our study, the rate of adenomas with irregular margins and heterogeneous echogenicity was higher than in previous studies, indicating that atypical features are more frequent in cases undergoing PTH-WO (35). In such cases, the PTH-WO test offers significant diagnostic support. Adenomas generally exhibit greater vascularity than thyroid nodules and lymph nodes. On Doppler ultrasonography, they typically appear as structures supplied by a polar artery and show a peripheral vascular pattern, which increases diagnostic sensitivity (36, 37). Although the literature reports polar vascularity in 97.6% of cases, our study revealed a lower rate (38), which may be related to the frequency of small, cystic, or ectopic adenomas. Furthermore, although adenomas with pronounced vascularity and irregular margins were observed to be larger, there were no significant differences in PTH-WO levels among these groups. This suggests that PTH-WO levels may be influenced not only by lesion size but also by additional factors such as hemodynamic structure, cellular composition, and fibrosis.

The PTH-WO test offers several notable advantages over other diagnostic methods that are costly and less accessible, including its high sensitivity for lesion localization, its ability to guide surgical treatment, and its utility in providing tissue confirmation for minimally invasive alternatives such as thermal ablation. These advantages, along with the method’s limitations, are summarized in **Table 4**. In this context, improving the understanding of the test and interpreting PTH-WO levels in conjunction with the morphological characteristics of the lesion may enhance its diagnostic reliability and clinical utility. This study highlights the diagnostic value of the PTH-WO test as a complementary tool for the preoperative localization of parathyroid adenomas, particularly in cases with inconclusive or discordant imaging findings. Our findings demonstrate that adenoma size and the presence of cystic components significantly influence PTH-WO levels, emphasizing the importance of incorporating these morphological parameters into the interpretation of test results and clinical decision-making. Moreover, our results indicate that the test may remain diagnostically reliable even at lower PTH-WO levels. On the other hand, the observation of unexpectedly low PTH-WO values in some large adenomas with irregular margins, heterogeneous echotexture, and prominent vascular features suggests that the test may be influenced not only by size-related features but also by underlying histopathological characteristics such as cellular architecture and fibrosis.

**Table 4:** Advantages and limitations of the PTH-WO method in clinical practice.

Advantages	Limitations
Provides biochemical confirmation with high specificity	There is no standardized diagnostic threshold
Contributes to diagnosis in cases with inconclusive imaging	Negative results do not exclude diagnosis; there is a risk of false negatives
Minimally invasive and generally a safe procedure	Requires caution due to risks of hematoma, fibrosis, and parathyromatosis
Assists in surgical planning and may prevent unnecessary interventions	Technical application requires operator experience
May reduce the need for costly advanced imaging methods	Plasma admixture during aspiration may complicate interpretation
Assists in the differential diagnosis of cervical structures (thyroid nodule/lymph node)	Deep, small lesions and coexisting thyroid pathologies may reduce aspiration efficiency

This table was prepared based on the findings of our study and relevant literature (5, 6, 8, 11, 12, 21–23).

Among the strengths of this study are the evaluation of a large patient cohort and the detailed analysis based on demographic and ultrasonographic findings. However, the single-center and retrospective design may limit generalizability due to factors such as patient selection, data loss, measurement errors, and the operator dependency of ultrasonography. In addition, due to the laboratory’s upper detection limit of 5,000 pg/mL, higher PTH-WO levels could not be measured, which imposed limitations on analysis and interpretation. The inclusion of only patients with non-localizable lesions on imaging introduces selection bias, meaning that the findings may apply not to the general PHPT population but rather to a selected group with difficult preoperative localization. Moreover, since all adenomas were PTH-WO positive and surgically confirmed, we were unable to perform receiver operating characteristic analysis to determine diagnostic thresholds by lesion size. While PTH-WO levels were compared with lesion size in solid adenomas, analysis was not feasible in cystic adenomas due to their small number and generally larger size. Lastly, although the threshold value of 33 pg/mL appears diagnostically relevant in our study, the small number of patients with PTH-WO levels below plasma PTH prevents the recommendation of a reliable threshold for this group. Therefore, this finding needs to be validated in larger patient populations.

In conclusion, PTH-WO is a valuable tool in enhancing the diagnostic accuracy of parathyroid adenomas; however, its reliability is influenced by various factors, including aspiration technique and the biological and morphological characteristics of the adenoma. Our study demonstrated a significant association between adenoma size and PTH-WO levels, particularly highlighting higher levels in adenomas with cystic components or large dimensions. Nonetheless, the limited effect of B-mode ultrasonographic features and vascular structure on PTH-WO levels suggests that the test should be interpreted not only in conjunction with anatomical evaluation but also in light of histological data. Future studies should include larger patient populations to determine more reliable cut-off values for PTH-WO, minimize technical limitations, and better understand the underlying biological variability.

## Data Availability

The datasets presented in this study can be found in online repositories. The names of the repository/repositories and accession number(s) can be found in the article/**Supplementary Material**.
