# Supplementary material for: Lesion size and cystic morphology are key determinants of parathyroid hormone washout in primary hyperparathyroidism
Source: Front Endocrinol (Lausanne). 2025 Sep 16;16:1654110. doi: 10.3389/fendo.2025.1654110 (PMC12479290; doi:10.3389/fendo.2025.1654110)
Supplement: Supplementary file 1 [file Table1.docx]

| **Supplementary Table 1. Comparison of PTH-WO Values Based on Clinical and Ultrasonographic Features** | | | | |
| --- | --- | --- | --- | --- |
| **Variable** | **n = 128^1^** | **PTH-WO (pg/mL)^2^** | **p-value*** | |
| **Sex** |  |  |  | |
| Male | 33 (25.78%) | 3,282.0 (557.0–5,000.0) | 0.07 | |
| Female | 95 (74.22%) | 1,428.0 (506.50–4,458.50) |  | |
| **Age** |  |  |  | |
| ≤50 years | 47 (36.72%) | 1,159.0 (356.50–4,861.50) | 0.25 | |
| >50 years | 81 (63.28%) | 2,039.0 (557.0–5,000.0) |  | |
| **BMI (kg/m²)** |  |  |  | |
| ≤30 | 50 (39.06%) | 2,664.0 (377.75–5,000.0) | 0.13 | |
| >30 | 78 (60.94%) | 1,403.0 (562.25–3,660.75) |  | |
| **Adenoma long diameter (mm)** |  |  |  | |
| ≤10 mm | 35 (27.34%) | 781.0 (339.50–1,721.50) | **0.005** | |
| >10 mm | 93 (72.66%) | 2,592.0 (580.0–5,000.0) |  | |
| **Adenoma short diameter (mm)** |  |  |  | |
| ≤5 mm | 32 (25%) | 890.0 (339.25–1,569.75) | **0.008** | |
| >5 mm | 96 (75%) | 2,599.50 (538.25–5,000) |  | |
| **Adenoma localization** |  |  |  | |
| Orthotopic | 108 (84.38%) | 1,379.50 (494.50–5,000.0) | 0.077 | |
| Ectopic | 20 (15.63%) | 3,949.50 (1,256.75–5,000.0) |  | |
| **B-mode features** |  |  |  | |
| **Echogenicity** |  |  |  | |
| Hypoechoic | 77 (60.16%) | 1,746.0 (557.0–5,000.0) | 0.99 | |
| Mixed echo | 51 (39.84%) | 1,617.0 (308.0–5,000.0) |  | |
| **Border regularity** |  |  |  | |
| Regular | 82 (64.06%) | 1,138.50 (362.0–5,000.0) | 0.22 | |
| Irregular | 46 (35.94%) | 2,438.50 (837.25–4,930.75) | |  |
| **Lobe count** |  |  |  | |
| Single | 109 (85.16%) | 1,651.0 (493.0–5,000.0) | 0.88 | |
| Bilobed | 19 (14.84%) | 1,939.0 (766.50–3,714.50) |  | |
| **Cystic component** |  |  |  | |
| Solid | 98 (76.56%) | 1,262.50 (484.75–4,596.50) | **0.02** | |
| Cystic | 30 (23.44%) | 3,892.50 (1237.0–5,000.0) |  | |
| **Doppler features** |  |  |  | |
| **Polar vascularity** |  |  |  | |
| Present | 100 (78.13%) | 1,416.0 (512.25–5,000.0) | 0.24 | |
| Absent | 28 (21.88%) | 2,846.50 (695.75–5,000.0) |  | |
| **Vascular arc** |  |  |  | |
| Present | 51 (39.84%) | 2,592.0 (657.0–5,000.0) | 0.24 | |
| Absent | 77 (60.16%) | 1,159.0 (361.0–5,000.0) |  | |
| **Diffuse vascularity** |  |  |  | |
| Present | 11 (8.59%) | 1,746.0 (667.50–3,803.50) | 0.89 | |
| Absent | 117 (91.41%) | 1,651.0 (493.0–5,000.0) |  | |
| ¹Presented as number (percentage), ²Presented as median (25%–75%), *Welch’s t-test.  PTH-WO, **parathyroid hormone** washout; BMI, body mass index; | | | | |
